# Transparent polycrystalline cubic silicon nitride

**DOI:** 10.1038/srep44755

**Published:** 2017-03-17

**Authors:** Norimasa Nishiyama, Ryo Ishikawa, Hiroaki Ohfuji, Hauke Marquardt, Alexander Kurnosov, Takashi Taniguchi, Byung-Nam Kim, Hidehiro Yoshida, Atsunobu Masuno, Jozef Bednarcik, Eleonora Kulik, Yuichi Ikuhara, Fumihiro Wakai, Tetsuo Irifune

**Affiliations:** 1 Deutsches Elektronen-Synchrotron DESY, Notkestr. 85, 22607, Hamburg, Germany; 2 Institute of Engineering Innovation, The University of Tokyo, 2-11-16 Yayoi, Bukyo-ku, Tokyo, 113-8656, Japan; 3 Geodynamics Research Center, Ehime University, 2-5 Bukyo-cho, Matsuyama, 790-8577, Japan; 4 Bayerisches Geoinstitut, Universität Bayreuth, 95440, Bayreuth, Germany; 5 National Institute for Materials Sciences, 1-1 Namiki, Tsukuba, Ibaraki 305-0044, Japan; 6 Graduate School of Science and Technology, Hirosaki University, 3 Bunkyo-cho, Hirosaki, 036-8561, Japan; 7 Laboratory for Materials and Structures, Tokyo Institute of Technology, R3-23, 4259 Nagatsuta-cho, Midori-ku, Yokohama, 226-8503, Japan; 8 Earth-Life Science Institute, Tokyo Institute of Technology, 2-12-1-1E-1 Ookayama, Meguro-ku, Tokyo 152-8500, Japan

## Abstract

Glasses and single crystals have traditionally been used as optical windows. Recently, there has been a high demand for harder and tougher optical windows that are able to endure severe conditions. Transparent polycrystalline ceramics can fulfill this demand because of their superior mechanical properties. It is known that polycrystalline ceramics with a spinel structure in compositions of MgAl_2_O_4_ and aluminum oxynitride (γ-AlON) show high optical transparency. Here we report the synthesis of the hardest transparent spinel ceramic, i.e. polycrystalline cubic silicon nitride (c-Si_3_N_4_). This material shows an intrinsic optical transparency over a wide range of wavelengths below its band-gap energy (258 nm) and is categorized as one of the third hardest materials next to diamond and cubic boron nitride (cBN). Since the high temperature metastability of c-Si_3_N_4_ in air is superior to those of diamond and cBN, the transparent c-Si_3_N_4_ ceramic can potentially be used as a window under extremely severe conditions.

There are two known polymorphs of Si_3_N_4_ at ambient pressure: α- and β-Si_3_N_4_ with hexagonal structures. The latter requires higher temperatures than the former to be synthesized. Practically, most Si_3_N_4_ polycrystalline materials are fabricated using α-Si_3_N_4_-rich powders and a part of the α-Si_3_N_4_ transforms into β-Si_3_N_4_ during sintering. In the sintered bodies, the β-Si_3_N_4_ grains frequently have elongated shapes with aspect ratios comparable to whiskers that act to toughen the material^1^. Therefore, the Si_3_N_4_ sintered bodies are whisker-reinforced ceramics, which makes this material useful in industry as parts of reciprocating engines, ball bearings, and cutting tools^1^. In these two phases, silicon atoms are fourfold (tetrahedrally) coordinated by nitrogen atoms.

c-Si_3_N_4_, which is often called γ-Si_3_N_4_, is stable under high pressure with a spinel structure^2^. In this structure, two thirds of the silicon atoms are sixfold (octahedrally) coordinated and the rest are fourfold coordinated. The sixfold coordination results in a significantly closer atomic packing and density almost 26% higher than those of the hexagonal phases^3^. c-Si_3_N_4_ is known to be synthesized above ~13 GPa (ref. 3) and has been a candidate as a superhard material^4^,^5^ that has a Vickers hardness value above 40 GPa. However, the mechanical properties have not yet been well-constrained.

In this article, we report the synthesis of the bulk polycrystalline form of c-Si_3_N_4_ with a grain size of ~150 nm. Using the sintered samples, all the elastic moduli (bulk, shear, Young’s modulus, and Poisson’s ratio) were determined; Vickers hardness and fracture toughness were measured. Surprisingly, the obtained bulk polycrystalline form exhibits high optical transparency. Atomic-resolution scanning transmission electron microscopy (STEM) observations were performed to elucidate the mechanism to make this material transparent.

## Results

We carried out synthesis runs at three different temperatures of 1600, 1700, and 1800 °C at a fixed pressure of 15.6 GPa. The starting material was commercially available α-Si_3_N_4_ powder whose oxygen content is less than 2 wt%. The phases present in the recovered samples were examined by X-ray diffraction (XRD) measurements. The sample synthesized at 1600 °C consists mainly of c-Si_3_N_4_ but the XRD peaks of α- and β-Si_3_N_4_ were also observed. The presence of the hexagonal phases may be explained by a kinetic hindrance of the transformation to c-Si_3_N_4_ at relatively lower temperature. This recovered sample was loosely sintered. The samples synthesized at 1700 and 1800 °C consist of a single phase of c-Si_3_N_4_ and they are well-sintered. These samples look translucent and transparent, respectively.

We determined a unit cell parameter of c-Si_3_N_4_ using an XRD pattern of a sample synthesized at 1800 °C: *a* = 7.7373 ± 0.0006 Å, which is consistent with that determined in a previous study (*a* = 7.7381 ± 0.0002 Å)^3^. The bulk density of this polycrystalline sample was measured to be 4.07 ± 0.08 g/cm^3^, which is also consistent with a theoretical density^3^ of 4.022 g/cm^3^. This result supports the fact that the sample consists of a single phase of c-S_i3_N_4_ and indicates that the porosity of this sintered sample is negligibly small. In addition, we measured the bulk chemical composition of this sample. The values we obtained are as follows: Si, 60.1 ± 0.3 wt%; N, 37.1 ± 0.4 wt%; O, 2.5 ± 0.2 wt%; Total, 99.7 ± 0.5 wt%. These values are very close to the calculated values for the stoichiometric Si_3_N_4_ (Si, 60.06 wt%; N, 39.94 wt%) and an oxygen-bearing α-phase^6^, Si_11.5_N_15_O_0.5_ (Si, 59.69 wt%; N, 38.83 wt%; O, 1.48 wt%).

**Figure 1:**
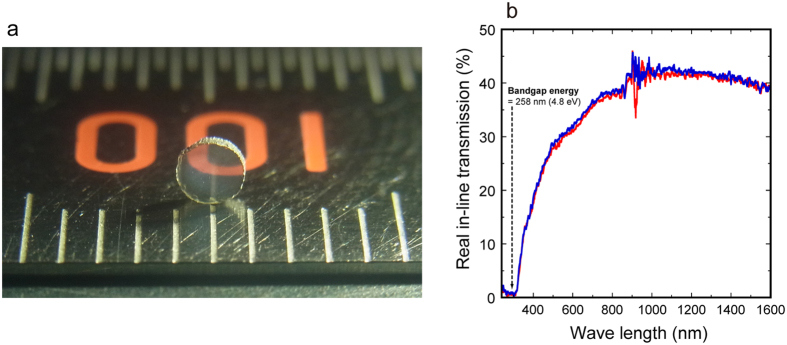
Transparent polycrystalline cubic silicon nitride. (**a**) A photograph of a bulk nanocrystalline form of c-Si_3_N_4_ synthesized at 15.6 GPa and 1800 °C. The division of the ruler (this side) is 1 mm. The thickness of the sample is 0.464 mm. (**b**) Real in-line transmission as a function of wave length. More than ten measurements were performed by rotating the sample around the light axis and by turning the sample over (the polished surfaces were always perpendicular to the light axis) in order to confirm that there is no orientation dependence of the transmission. The red and blue lines show the two representative results.

Figure 1a shows a photograph of a recovered sample synthesized at 1800 °C, and it is transparent pale gray. A letter on a ruler behind the sample is clearly seen. Real in-line transmission^7^ (RIT) was measured as a function of wavelength (Fig. 1b). In these measurements, all light that is scattered at an angle >0.3° is not detected. Therefore, the RIT is a good measure for the visual transparency^7^. The RIT of this sample is 18–38% for visible light (wavelength of 400–800 nm). We can see the presence of a weak and broad absorption at a wavelength of 500–700 nm, which may cause the pale grey color of this sample. The very low RIT values up to 300 nm can be related to the bandgap energy of 258 nm ( = 4.8 ± 0.2 eV)^8^.

**Figure 2:**
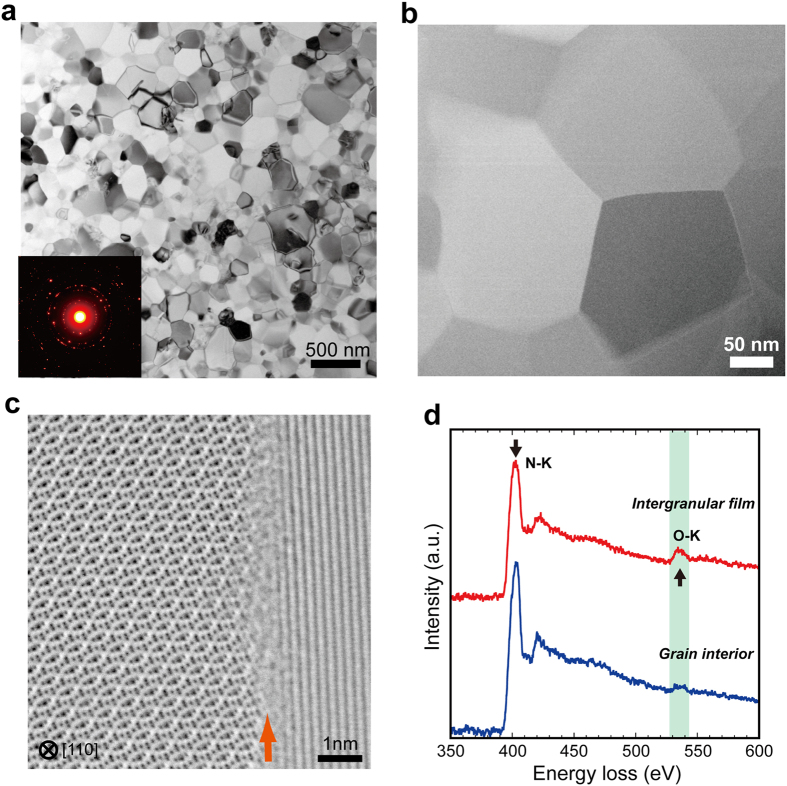
Conventional TEM and atomic-resolution STEM observations. (**a**) a bright-field TEM image of c-Si_3_N_4_ synthesized at 15.6 GPa and 1800 °C. The average grain size is 143 ± 59 nm with the maximum grain size of ~400 nm. The inset shows an electron diffraction pattern indicating that the grains are randomly oriented in this polycrystalline material. (**b**) a low-angle annular dark-field (LAADF) STEM image of a triple junction (the center of the image) showing that no pore and no triple pocket exists. (**c**) an annular bright-field scanning transmission electron microscopy (ABF-STEM) image at a disordered/amorphous IGF (indicated by the orange arrow) between two grains, where the left grain is viewed along the [110] orientation. (**d**) EELS spectra at an IGF (top) and in a grain interior (bottom). The decrease of the N-K edge peak and the increase of O-K edge peak were observed at the IGF.

We observed the microstructure of the transparent polycrystalline c-Si_3_N_4_. Figure 2a shows an example of the bright-field TEM images. The presence of equigranular texture was observed and the average grain size is 143 ± 59 (one standard deviation) nm. No residual pore and no triple pocket was observed at multi-grain junctions (Fig. 2b), which is consistent with the results of the density measurements. Most of the c-Si_3_N_4_ grains have straight grain boundaries and they are almost dislocation-free, which is consistent with the presence of sharp peaks in the XRD patterns (Supplementary Fig. S1). Atomic-resolution STEM observations at grain boundaries between two grains show the presence of disordered/amorphous intergranular films^9^ (IGFs) with a thickness of less than 1 nm (Fig. 2c). The results of electron energy-loss spectroscopy (EELS) measurements at the IGFs and grain interiors show that oxygen atoms preferentially segregate to the IGFs (Fig. 2d).

**Figure 3:**
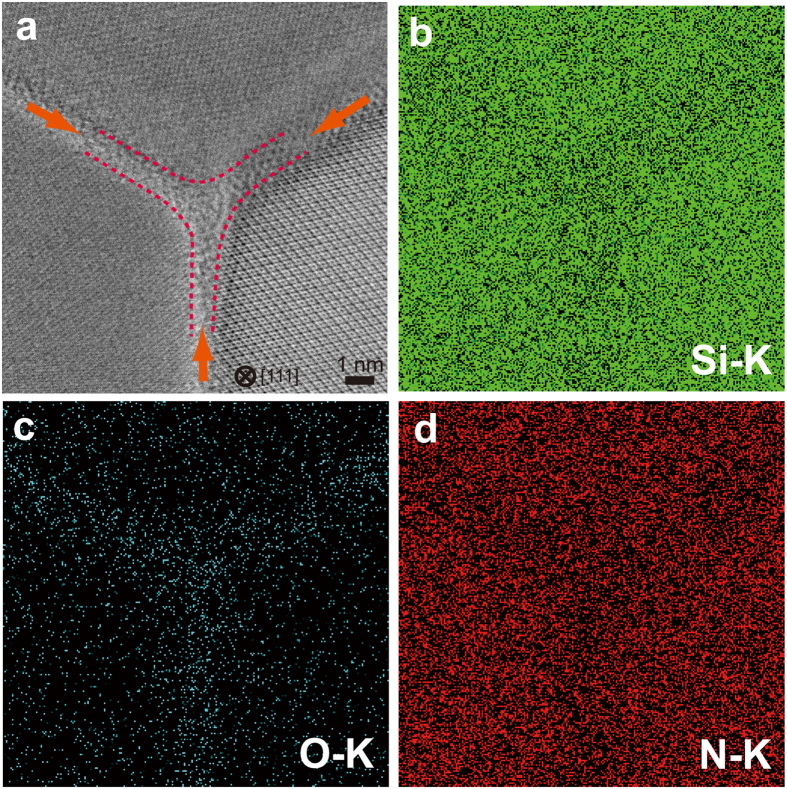
Atomic-resolution STEM observation and STEM-EDS chemical mappings at a triple junction area. (**a**) An ABF-STEM image of the triple junction area shown in Fig. 2b. The thickness of the IGFs near the triple junction is ~1 nm. No triple pocket exists in the atomic-scale observation. EDS chemical mappings of silicon (**b**), oxygen (**c**) and nitrogen (**d**). The results show that the IGFs consist of silicon oxynitride.

Atomic resolution STEM observations were also performed at multi-grain junctions (Fig. 3a). We observed that the thickness of IGFs near the multi-grain junctions (~1 nm) is similar to that between two grains, which causes the absence of triple pockets. Elemental mappings of silicon, nitrogen, and oxygen (STEM-EDS, energy dispersive X-ray spectroscopy) confirmed that oxygen atoms segregate to the IGFs and revealed that the IGFs consist of silicon oxynitride (Fig. 3b–d).

The very thin IGFs and the absence of triple pockets could be the reasons for the optical transparency of the bulk polycrystalline c-Si_3_N_4_. Scattering of incident light is caused by the following factors^10^: (1) optically inhomogeneous materials with secondary phases that have different refractive indices; (2) not isotropic crystals by birefringence. The absence of triple pockets (no second phase) and the cubic spinel structure (MgAl_2_O_4_ spinel^11^ and γ-AlON (ref. 12), which are known as popular transparent ceramics, have the same crystal structure) causes small scattering losses resulting in the optical transparency of bulk polycrystalline c-Si_3_N_4_.

Although α- and β-Si_3_N_4_ phases are intrinsically transparent because of the wide bandgaps^13^ (single crystals of α-Si_3_N_4_ were reported to be transparent)^14^, the Si_3_N_4_ sintered compacts that consist of α- and β-phases look opaque^1^. In the case of the sintering at lower pressures (e.g. hot-pressing), sintering aids such as MgO, SiO_2_, and Al_2_O_3_ are required to obtain the toughened and highly densified compacts^1^. For example, MgO reacts with silicon and oxygen in the system and the magnesium silicate is molten under high temperature; after cooling, it remains mainly in triple pockets as an amorphous phase^1^. Even in the case of sintered β-Si_3_N_4_ ceramics without additives, the presence of amorphous triple pockets was observed^15^ and their dimensions are several tens to several hundreds of nanometers^16^. The chemical composition of the β-Si_3_N_4_ grains is nearly pure Si_3_N_4_ and that of the amorphous triple pockets is SiO_2_ with a small amount of nitrogen^16^. Since the β-Si_3_N_4_ cannot incorporate the impurities into its structure, the formation of the triple pockets may be inevitable as reservoirs of additives and impurities in the sintered bodies. The refractive index of the amorphous phase in the triple pockets can be different from those of the crystalline phases (α- and β-phase with hexagonal symmetry), which causes scattering of the incident light resulting in opaque sintered bodies.

On the other hand, it has been reported that some polycrystalline α-SiAlONs exhibit optical transparency^17^. These materials are isostructural with α-Si_3_N_4_, in which silicon and nitrogen are partially substituted by aluminum and oxygen (Si_3_N_4_-based solid solutions)^17^. The incorporation of the additives into the structure produces little or no triple pocket, which causes small scattering losses of light resulting in optical transparency. This mechanism may be applied to the case of the transparent c-Si_3_N_4_. The absence of triple pockets in this material (Fig. 3a) indicates that at least a part of the oxygen impurities is incorporated into the c-Si_3_N_4_ structure.

The composition of the hypothetical oxygen-bearing c-Si_3_N_4_ may be inferred by analogy from that of α-Si_3_N_4_. It was reported that α-Si_3_N_4_ contains oxygen in its crystal structure, which could be explained by nitrogen vacancies with electrical neutrality being obtained by an appropriate number of silicon vacancies or Si^3+^ species^1^. The compositions suggested are Si_23_N_30_O or Si^4+^_20_Si^3+^_4_N_30_O, respectively, which are similar to the compositions of the starting material and the transparent c-Si_3_N_4_ ceramic. In addition, aluminum oxynitrides with a spinel structure (γ-AlONs) contain cation vacancies^18^. For example, in Al_23_O_27_N_5_ spinel, 8 Al^3+^ occupy all the tetrahedral sites and 15 Al^3+^ occupy octahedral sites with 1 vacancy^18^. Thus, the cation vacancy model (Si_23_N_30_O) might be preferable for the hypothetical oxygen-bearing c-Si_3_N_4_. Actually, our EELS spectrum at grain interior might show the presence of oxygen in the structure (Fig. 2d). The silicon and nitrogen vacancies might cause the pale grey color of this transparent c-Si_3_N_4_ (Fig. 1a). Further studies are required to elucidate the mechanism of oxygen incorporation into c-Si_3_N_4_ and the resultant optical transparency.

In order to show a potential of the transparent c-Si_3_N_4_ ceramic as a hard and tough window, the mechanical properties of this material were measured. Since it is optically transparent, Brillouin scattering was performed to measure longitudinal and transverse wave velocities (*V*_P_ and *V*_S_, respectively). We obtained *V*_P_ = 12.55 km/s and *V*_S_ = 7.84 km/s with estimated errors of 0.5%. Then, we can calculate all the elastic moduli at ambient conditions using the theoretical density^3^ of 4.022 g/cm^3^: bulk modulus (*B*), 303.4 ± 4.0 GPa; shear modulus (*G*), 247.5 ± 1.0 GPa; Young’s modulus (*E*), 583.8 ± 10.1 GPa; Poisson’s ratio (ν), 0.1793 ± 0.0056. The comparison with previous results^5^,^19^,^20^,^21^,^22^ is shown in Table 1.

**Figure 4:**
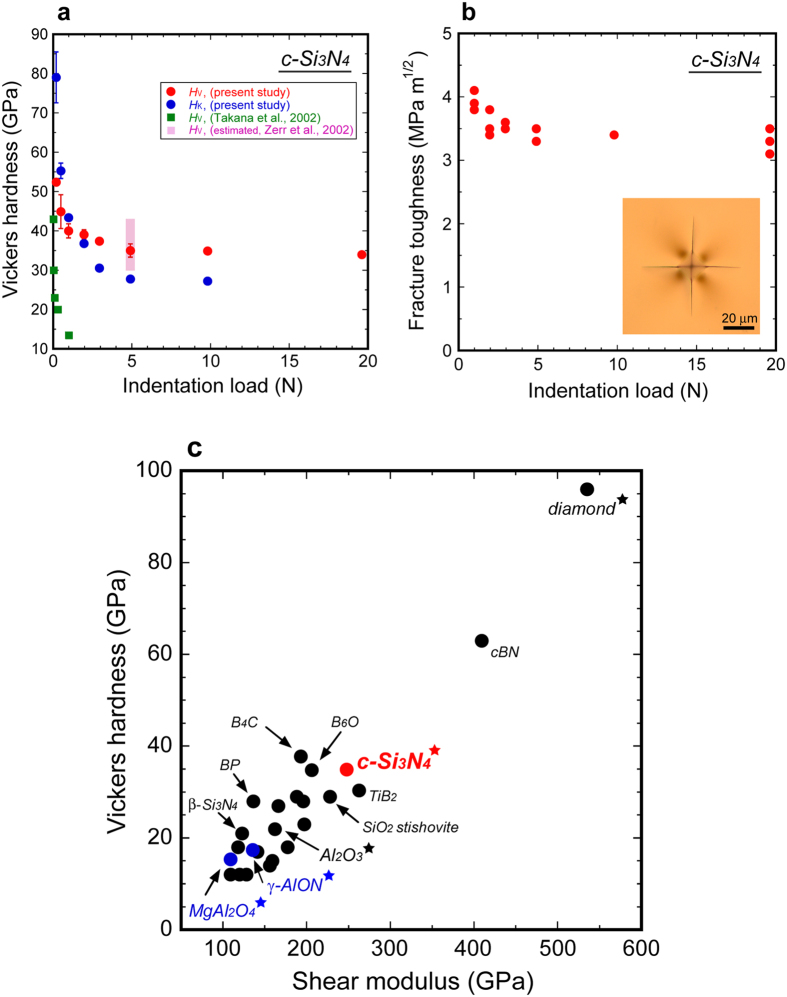
Mechanical properties of cubic silicon nitride. (**a)** Indentation load dependence of Vickers (*H*_V_) and Knoop hardness (*H*_K_) of c-Si_3_N_4_. Previous results are shown for comparison (squares). (**b**) Indentation load dependence of fracture toughness (*K*_Ic_). The inset shows a micrograph of a representative indentation trace with cracks at an indentation load of 9.8 N (by transmitted light microscopy). Since the sample is optically transparent, we can observe radial-median cracks propagated into the sample. (**c**) *H*_V_ vs. shear modulus (*G*) for hard materials (closed circles). c-Si_3_N_4_ is in the group of the third hardest material with B_4_C and B_6_O. c-Si_3_N_4_ is much harder than MgAl_2_O_4_-spinel and γ-AlON. The polycrystalline materials that exhibit optical transparency are shown with stars.

Vickers indentation tests were carried out at indentation loads between 0.196 and 19.6 N to measure the Vickers hardness (*H*_V_) and evaluate the fracture toughness (*K*_Ic_). Fig. 4a shows the indentation load dependence of *H*_V_. The *H*_V_ decreases with indentation load to an asymptotic value beyond 5 N. We employed *H*_V_ = 34.9 ± 0.7 GPa at 9.8 N as a representative value of this material. The comparison with previous results^4^,^5^ is also shown in this plot. 6-Mar-2017 11:376-Mar-2017 11:376-Mar-2017 11:376-Mar-2017 11:376-Mar-2017 11:376-Mar-2017 11:37 4b shows an indentation load dependence of *K*_Ic_. The average of all the measured values is 3.5 ± 0.2 MPa·m^1/2^. The transparent c-Si_3_N_4_ is tougher than transparent MgAl_2_O_4_ spinel (*K*_Ic_~1.7 MPa·m^1/2^)^23^ and γ-AlON (*K*_Ic_~2.4 MPa·m^1/2^)^23^, and is as tough as polycrystalline alumina, which is a popular structural ceramic in industry. The obtained mechanical properties and comparison with previous results^4^,^5^,^20^,^21^,^22^,^24^,^25^ are also shown in Table 1.

In order to compare the hardness of c-Si_3_N_4_ with those of other materials, we have updated a plot proposed by Teter^26^, *H*_V_ vs. *G* (Fig. 4c, the details are shown in supplementary information). c-Si_3_N_4_ is as hard as and elastically stiffer than B_4_C and B_6_O. These three materials are in the group of the third hardest materials (not superhard materials) next to diamond and cBN.

## Discussion

The polycrystalline c-Si_3_N_4_ shows intrinsic transparency (Fig. 1b): no strong light absorption at wavelengths below its bandgap energy. Diamond is the hardest material and the synthesis of transparent nano-polycrystalline diamond (NPD) was reported^27^. NPD shows its characteristic brownish color, which is given by a continuous absorption at wavelengths between 500 and 700 nm. This absorption could be attributed to lattice defects induced by plastic deformation during the synthesis process^28^. This material also shows strong continuous absorption at wavelengths between 225 nm (band gap energy) and 450 nm, which is attributed to electron transitions from nitrogen (impurity) to the conduction band^28^. Thus, NPD is transparent, but does not show its intrinsic transparency. Polycrystalline cBN materials have been reported to be translucent^29^; B_4_C and B_6_O materials are opaque. Thus, the polycrystalline c-Si_3_N_4_ is nearly color-less and the second hardest ceramic window (Fig. 4c).

The transparent polycrystalline c-Si_3_N_4_ can potentially be employed in industry to protect optical sensors and detectors under severe conditions. One of the advantages of this material is an excellent thermal metastability in air^24^, at least up to 1400 °C, which is superior to that of diamond, cBN, and other hard materials^30^. On the other hand, one of the limitations of this material for industrial applications is the small size of the bulk material because of synthesis under high pressure above 13 GPa (ref. 3). In this article, we reported the synthesis of the transparent disks with diameter of ~2 mm (Fig. 1a). If one of the world’s largest high-pressure instruments is employed for the synthesis, transparent c-Si_3_N_4_ disks with diameter larger than 10 mm can be synthesized^31^. The centimeter-sized transparent disks can be used in industry as windows for small devices.

## Methods

### Starting material and the container for synthesis under high pressure and temperature

The starting power was commercially available α-Si_3_N_4_ powder (SN-E10, Ube Industries, Ltd., Ube, Japan). According to the product catalog, the proportion of the α-phase is >95 wt%; the oxygen content is <2 wt%.; the specific surface area is 9–13 m^2^/g. The as-received powder was dried in an oil-free vacuum oven (~8 hPa) at 200 °C for ~12 hours. The dried powder was enclosed in a sample capsule made of a platinum sleeve and disks, which were embedded into an outer MgO sleeve with MgO lids. Before loading the sample, the platinum and MgO parts were heated at 1000 °C for ~10 min. After loading the sample, the assembled sample container was dried again in the vacuum oven at 150 °C for more than 2 hours before the synthesis runs.

### Synthesis under high-*PT*

High-pressure and high-temperature synthesis experiments were performed using a Kawai-type apparatus, installed at DESY, with a Walker-module (mavo press LPR 1000-400/50; Max Voggenreiter GmbH, Mainleus, Germany). The maximum force of this instrument is 1000 tonf. The second stage anvils were tungsten carbide cubes with a truncated edge length of 7 mm. A Cr_2_O_3_-doped magnesia octahedron with an edge length of 14 mm was used as the pressure transmitting medium. A cylindrical LaCrO_3_ furnace (Nikkato Corp., Osaka, Japan) was employed. A sample container made of platinum and MgO was embedded into the furnace. Further details are shown in Supplementary information.

### XRD measurements

XRD patterns of the recovered samples were obtained using an X-ray diffractometer with Cu-Kα radiation (MiniFlex-600; Rigaku, Japan). The measurements were also carried out at the P02.1 in PETRA III, Germany. X-rays monochromatized by a (111) diamond and a (111) silicon crystal in Laue geometry with an energy of ~60 keV were used. The incident beam-size was 300 × 300 μm^2^. The transmission geometry and a two-dimensional detector (XRD 1621, PerkinElmer) were employed. The sample-to-detector distance (~900 mm) and the detector orthogonality were calibrated using the diffraction pattern for cerium dioxide powder.

### Bulk density and chemical composition measurements

Density of a recovered sample was measured with an AccuPyc II 1340 pycnometer, using He gas displacement and mass measurements. Chemical composition of the recovered sample was measured using a scanning electron microscope equipped with an energy-dispersive detector. The accelerating voltage, current, and working distance was 20 kV, 1 nA, and 10 mm, respectively. During the data collection, the electron beam was scanned over the surface of the sample in the area of ~1 μm × ~1 μm. Data aquisition time was 20 s. The standards employed for elemental quantifications were as follows: Si, SiC; N, cBN; O, SiO_2_. The polished surface of the recovered sample was coated with osmium.

### Light transmission measurements

Real in-line transmission of a recovered sample was measured in the wavelength range of 240 and 1600 nm using a double-beam spectrophotometer (SolidSpec-3700DUV, Shimadzu, Japan), installed at NIMS, equipped with an integrating sphere. The diameter of light at the sample is about 0.8 mm and an aperture with diameter of 2 mm was used at the entrance of the integrating sphere. The distance between the sample and the aperture is 550 mm. The top and bottom surfaces of the disk-shape sample were polished using diamond pastes down to 1 μm and the thickness is 0.464 mm.

### TEM observations

For conventional BF-TEM and electron diffraction observations were carried out by JEM 2010HC (JEOL Ltd.). For atomic-resolution imaging and spectroscopy, we used an aberration-corrected STEM of ARM 300CF (JEOL Ltd.), installed at University of Tokyo, equipped with a JEOL ETA corrector, a cold field-emission gun, Gatan Quantum ER EELS spectrometer, and a JEOL silicon drift detector for EDX spectrometer. The used illumination semi-angle is 24 mrad and the ABF- and ADF-detector is spanning 12–24 and 80–200 mrad, respectively. We used dual-EELS mode to simultaneously acquire core-loss and zero-loss spectra (the energy dispersion is 0.25 eV per channel) and the energy drift was corrected via post processing. We operated the STEM at 300 kV with typically 20 pA beam current, where no significant beam damage was observed. The grain size of the sample was determined by direct measurement of 100 grains that appear black (satisfying the Bragg condition) in a conventional BF-TEM image^32^.

### Brillouin scattering measurements

Elastic wave velocities were measured using the Brillouin system, installed at University of Bayreuth, employing 532 nm laser light, a multi-pass tandem Fabry-Perot interferometer and an avalanche photodiode for signal detection. Measurements were performed in symmetric forward scattering geometry using a scattering angle of 80°. Spectrum collection times were about 5 minutes at a laser power of 70 mW.

### Hardness and fracture toughness measurements

*H*_V_ was measured using a microhardness indenter (HM-221, Mitsutoyo) installed at DESY. A hard steel standard with 900 HV was used. The holding time under the indentation load is 15 s. *H*_V_ was calculated using the following equation: *H*_V_ = 1854.4 *P*/*d*^2^, where *P* is the applied load (N) and d is the arithmetic mean of the two diagonals (μm) of a Vickers indentation trace. *K*_Ic_ was calculated from the crack length, *c*, using the following equation: *K*_Ic_ = ξ (*E*/*H*_V_)^1/2^ (*P*/*c*^3/2^), where *E* is the Young’s modulus (GPa) and ξ is a calibration constant of 0.016 (ref. 33). Knoop hardness (*H*_K_) was measured using the same instrument. The applied load was 0.196–9.8 N with a holding time of 15 s.

## Additional Information

**How to cite this article**: Nishiyama, N. *et al*. Transparent polycrystalline cubic silicon nitride. *Sci. Rep.* **7**, 44755; doi: 10.1038/srep44755 (2017).

**Publisher's note:** Springer Nature remains neutral with regard to jurisdictional claims in published maps and institutional affiliations.

## Supplementary Material

Supplementary Information

## Figures and Tables

**Table 1:** Mechanical properties of cubic silicon nitride

Reference	*B* (GPa)	*G* (GPa)	*E* (GPa)	ν	*H*_V_ (GPa)	*K*_Ic_ (MPa·m^1/2^)
*Experimental*						
Present study	303.4 (40)	247.5 (10)	583.8 (101)	0.1793 (56)	34.9 (7)	3.5 (2)
Soignard *et al*.^19^	308 (5)	—	—	—	—	—
Zerr *et al*.^5^	290 (5)	148 (16)^*^	—	—	30–43^**^	—
Jiang *et al*.^24^	—	—	—	—	35.31^†^	—
Tanaka *et al*.^4^	—	—	—	—	43–13^‡^	—
*Theoretical*						
Soignard *et al*.^19^	305	258.3	—	—	—	—
Dong *et al*.^20^	305	258.3	—	—	30	—
Kocer *et al*.^21^	310.9	264.6	—	—	47	—
He *et al*.^22^	313.9	272.9	—	—	33	—
Gao *et al*.^25^	—	—	—	—	30.9	—

*An estimated value from *B* and *E* determined by nanoindentation technique.

**A value at 5N estimated from the results obtained by nanoindentation measurements.

^†^No indentation load is shown in this report.

^‡^Values obtained at indentation loads between 10 mN and 1000 mN.
