# Supplementary material for: Transparent polycrystalline cubic silicon nitride
Source: Sci Rep. 2017 Mar 17;7:44755. doi: 10.1038/srep44755 (PMC5355983; doi:10.1038/srep44755)
Supplement: Supplementary Information [file srep44755-s1.pdf]

Supplementary information

Transparent polycrystalline cubic silicon nitride

Norimasa Nishiyama[1,9,*], Ryo Ishikawa[2], Hiroaki Ohfuji[3], Hauke Marquardt[4], Alexander Kurnosov[4], Takashi Taniguchi[5], Byung-Nam Kim[5], Hidehiro Yoshida[5], Atsunobu Masuno[6], Jozef Bednarcik[1], Eleonora Kulik[1,4], Yuichi Ikuhara[2], Fumihiro Wakai[7], Tetsuo Irifune[8]

1: Deutsches Elektronen-Synchrotron DESY, Notkestr. 85, 22607 Hamburg, Germany
2: Institute of Engineering Innovation, The University of Tokyo, 2-11-16 Yayoi, Bukyo-ku, Tokyo 113-8656, Japan
3: Geodynamics Research Center, Ehime University, 2-5 Bukyo-cho, Matsuyama 790-8577, Japan
4: Bayerisches Geoinstitut, Universität Bayreuth, 95440 Bayreuth, Germany
5: National Institute for Materials Sciences, 1-1 Namiki, Tsukuba, Ibaraki 305-0044, Japan
6: Graduate School of Science and Technology, Hirosaki University, 3 Bunkyo-cho, Hirosaki 036-8561, Japan
7: Laboratory for Materials and Structures, Tokyo Institute of Technology, R3-23, 4259 Nagatsuta-cho, Midori-ku, Yokohama 226-8503, Japan
8: Earth-Life Science Institute, Tokyo Institute of Technology, 2-12-1-1E-1 Ookayama, Meguro-ku, Tokyo 152-8500, Japan
9: Now at Laboratory for Materials and Structures, Tokyo Institute of Technology, 4259 Nagatsuta-cho, Midori-ku, Yokohama 226-8503, Japan

*email: nishiyama.n.ae@m.titech.ac.jp

This PDF file inclues:

1. **Pressure and temperature calibrations; *P*-*T* path of the synthesis runs**
2. **XRD patterns of the recovered samples**
3. **Chemical composition measurements**
4. **Sample preparation for TEM observations**
5. **Grain size distribution**
6. **Brillouin spectroscopy**
7. **Fracture surface observation**
8. **Numerical data for Fig. 4c to update the H_V-G plot for hard materials**

1. Pressure and temperature calibrations; *P-T* path of the synthesis runs:

Pressure was calibrated at room temperature using a fixed point of pressure: the semiconductor to metal transition of ZnS at 15.6 GPa. Temperature was calibrated in a separate run using a W5%Re/W26%Re thermocouple (C-type). For synthesis runs, a pressure of 15.6 GPa was applied first at room temperature for ~2 h and temperature was then increased at a constant load corresponding to this pressure. Temperature was increased to the target values in two steps: first, temperature was increased to 450°C with a heating rate of ~50°C/min; secondly, temperature was rapidly increased to the target value within 10 seconds. The target temperature was maintained for 30 min. Then temperature was gradually decreased to 400°C for ~10 min. Decompression at ~400°C took 3 h. After the decompression, the sample was recovered.

2. XRD patterns of the recovered samples:

Figure S1 shows synchrotron XRD patterns of polycrystalline c-Si_3N_4 samples synthesized at 1700 and 1800°C. These two patterns look alomost identical and all the diffracted peaks are explained by the presence of the single phase of c-Si_3N_4. The peaks look very sharp, indicating that no residual stress exists in the sintered bodies.

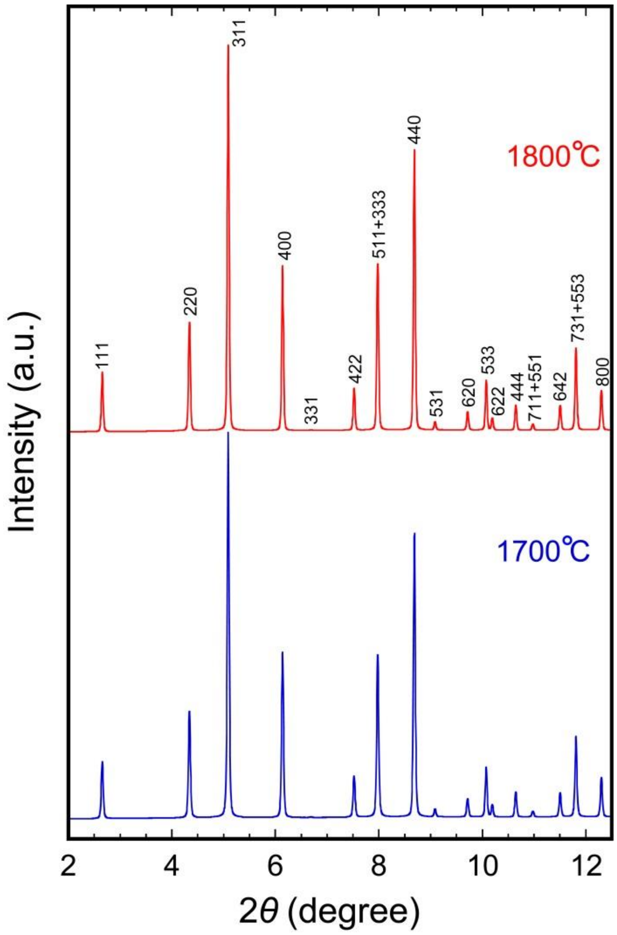

Figure S1. Synchrotron XRD patterns of bulk nanocrystalline forms of c-Si_3N_4 synthesized at 1700 and 1800°C under a fixed pressure of 15.6 GPa. Energy of the X-rays was about 60 keV.

3. Chemical composition measurements:

Figure S2 shows an example of energy-dispersive X-ray spectrum obtained from the sample synthesized at 15.6 GPa and 1800°C. We can see the presence of silicon, nitrogen, and oxygen; no other element was detected.

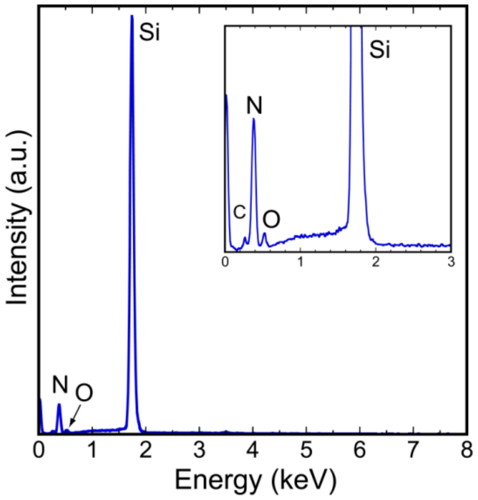

Figure S2. An example of EDS spectra of c-Si3N4 synthesized at 15.6 GPa and 1800°C. The presence of Si, N, and O can be seen. The calculated chemical composition is shown in the main text.

4. Sample preparation for TEM observations:

The electron transparent thin specimen was prepared by the following procedure: cutting the pellet sample, mechanical polished the sample less than 50 μm thickness, and a conventional Ar ion beam thinning method. We note that, to suppress Ar ion beam damages, the sample was cooled down to liquid nitrogen temperature.

5. Grain size distribution:

Figure S3 shows grain size distribution of the transparent c-Si_3N_4 polycrystalline sample synthesized at 15.6 GPa and 1800°C. We directly measured 100 grains that appear black (satisfying the Bragg condition) in a BF-TEM image[32].

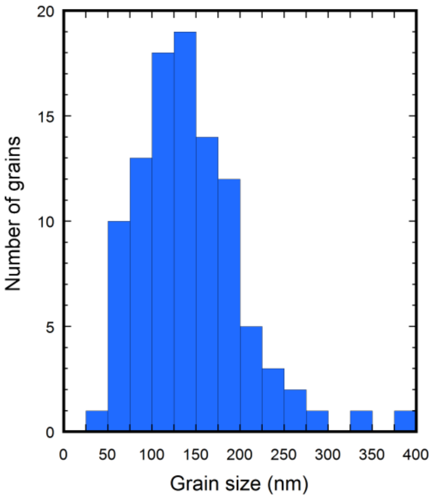

Figure S3. Grain size distribution of the transparent c-Si_3N_4 polycrystalline sample synthesized at 15.6 GPa and 1800°C. The average grain size is 143 ± 59 nm.

6. Brillouin spectroscopy:

Brillouin pectra collected in several positions on the polycrystalline sample as well as with varying phonon propagation directions showed that the sample is elastically isotropic as expected (less than 50 m/s deviation of velocities from the average velocity) becuase of the randomly oriented nanocrystalline c-Si_3N_4. Figure S4 shows an example of the spectra.

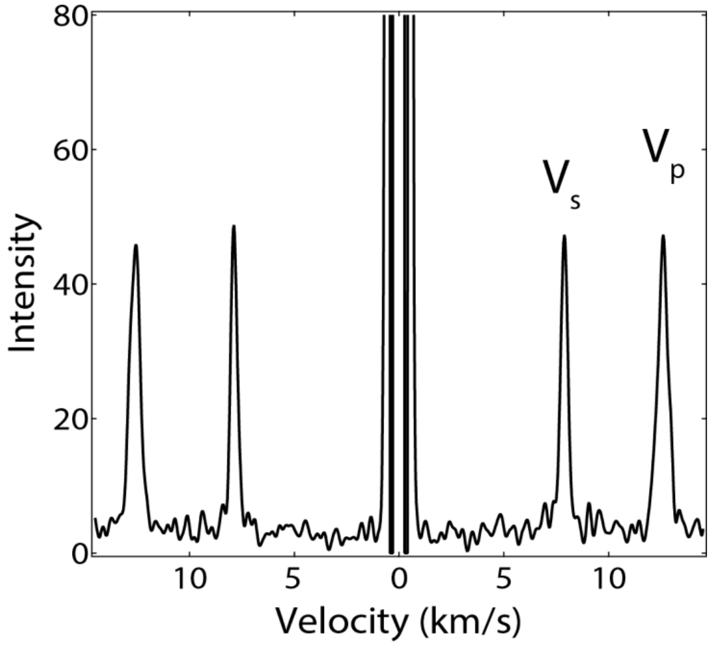

Figure S4. An example of Brillouin spectra of a bulk nanocrystalline form of c-Si_3N_4 synthesized at 15.6 GPa and 1800°C.

7. Fracture surface observation:

A field emission scanning electron microscope (JSM-7000F, JEOL) operating at 15 kV was used for secondary electron image observations of a fracture surface of a polycrystalline c-Si_3N_4 synthesized at 15.6 GPa and 1800°C. The fracture surface was coated with osmium. Figure S5 shows an example of secondary electron images of a fracture surface. This image clearly shows that the intergranular fracture is dominant in this sample.

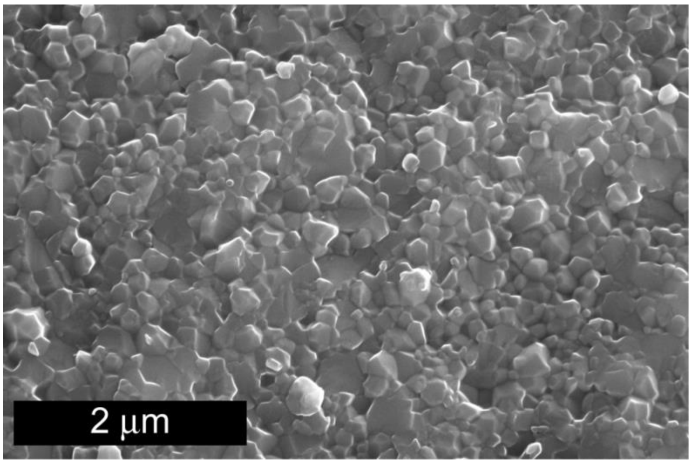

Figure S5. An example of secondary electron images of a fracture surface of a polycrystalline c-Si_3N_4.

8. Numerical data for Fig. 4c to update the H_V-G plot for hard materials:

Table S1 shows numerical data for the Fig. 4c. Other data are the same as those used by Teter[26]. Recently, synthesis of some potential superhard materials (H_V > 40 GPa), such as hetrodiamonds (c-BC_2N[S15] and c-BC_5[S17]) and a high-pressure phase of boron (γ-B_{28}[S18]), was reported, but the mechanical properties of these materials have been still under debate. Thus, at the moment, these materials are not included in the plot. Diamond and cBN are the well-established hardest and second hardest materials, respectively.

Table S1. H_V and G_0 values for Figure 4.

Material	H_V (GPa)	Reference	G_0 (GPa)	Reference
B_4C	37.8	[S1] Ji et al. 2015	193	[S2] Dodd et al. 2002
c-Si_3N_4	34.9	Present study	247.5	Present study
B_6O	34.8	[S3] Kleebe et al. 2008	206	[S4] Petrak et al. 1974
TiB_2	30.4	[S5] Rizzo et al. 1962	262.4	[S6] Spoor et al. 1997
SiO_2-stishovite	29	[S7] Nishiyama et al. 2014	228	[S8] Jiang et al. 2009
BP	28	[S9] Mukhanov et al. 2013	136.4	[S10] Wettling and Windscheif 1984
γ-AlON	17.4	[S11] Klement et al. 2008	135.5	[S12] Graham et al. 1988
$MgAl_2O_4$	15.4	[S13] Morita et al. 2009	109	[S14] Chopelas 1996
c-BC_2N	76	[S15] Solozhenko et al. 2001	238	[S16] Tkachev et al., 2003
c-BC_5	71	[S17] Solozhenko et al. 2009	-	-
γ-B_{28}	58	[S18] Zarechnaya et al. 2009	227.2	[S19] Qin et al., 2012

References:

[S1] Ji, W. et al. Sintering boron carbide ceramics without grain growth by plastic deformation as the dominant densification mechanism. *Sci. Rep.*, **5**, 15827 (2015)

[S2] Dodd, S. P. Saunders, G. A. & James, B. Temperature and pressure dependence of the elastic properties of ceramic boron carbide (B_4C). *J. Mater. Sci.*, **37**, 2731-2736 (2002).

[S3] Kleebe, H. –J., Lauterbach, S., Shabalala, T. C., Herrmann, M. & Sigalas, I. B_6O: a correlation between mechanical properties and microstructure evolution upon Al_2O_3 addition during hot pressing. *J. Am. Ceram. Soc.*, **91**, 569-575 (2008).

[S4] Petrak, D. R., Ruh, R. & Aktins, G. Mechanical properties of hot-pressed boron suboxide and boron. *Am. Ceram. Soc. Bull.*, **53**, 569-573 (1974).

[S5] Rizzo, H. F., Simmons, W. C. & Bielstein, H. O. The existence and formation of the solid B_6O. *J. Electrochemical Soc.*, **109**, 1079-1082 (1962).

[S6] Spoor, P. S., Maynard, J. D., Pan, M. J., Green, D. J., Hellmann, J. R. & Tanaka, T. Elastic constants and crystal anisotropy of titanium diboride. *Appl. Phys. Lett.*, **70**, 1959-1961 (1997).

[S7] Nishiyama, N. et al. Fracture-induced amorphization of polycrystalline SiO_2 stishovite: a potential platform for toughening in ceramics. *Sci. Rep.*, **4**, 6558 (2014).

[S8] Jiang, F., Gwanmesia, G. D., Dyuzheva, T. I. & Duffy, T. S. Elasticity of stishovite and acoustic mode softening under high pressure by Brillouin scattering. *Phys. Earth Plant. Inter.*, **172**, 235-240 (2009).

[S9] Mukhanov, V. A., Sokolov, P. S., Le Godec, Y. & Solozhenko, V. L. Self-propagating high-temperature synthsis of boron phosphide. *J. Superhard Mater.*, **35**, 415-417 (2013).

[S10] Wettling, W. & Windscheif, J. Elastic constants and refractive index of boron phosphide. *Solid State Comm.*, **50**, 33-34 (1984).

[S11] Klement, R., Rolc, S., Mikulikova, R. & Krestan, J. Transparent armour materials. *J. European Ceram. Soc.*, **28**, 1091-1095 (2008).

[S12] Graham, E. K., Munly, W. C., McCauly, J. W. & Corbin, N. D. Elastic properties of polycrystalline aluminu oxynitride spinel and their dependence on pressure, temperature, and composition. *J. Am. Ceram. Soc.*, **71**, 807-812 (1988).

[S13] Morita, K., Kim, B-N., Hiraga, K. & Yoshida, H. Fabrication of high-strength transparent $MgAl_2O_4$ sipnel polycrystals by optimizing spark-plasma-sintering conditions. *J. Mater. Res.*, **24**, 2863-2872 (2009).

[S14] Chopelas, A. The fluorescence sideband method for obtaining acoustic velocities at high compressions: application to MgO and $MgAl_2O_4$. *Phys. Chems. Minerals*, **23**, 25-37 (1996)

[S15] Solozhenko, V. L., Andrault, D., Fiquet, G., Mezouar, M. & Rubie, D. C. Synthesis of superhard cubic BC_2N. *Appl. Phys. Lett.*, **78**, 1385-1387 (2001).

[S16] S. N. Tkachev, V. L. Solozhenko, P. V. Zinin, M. H. Maghnani, L. C. Ming, *Phys. Rev. B*, **2003**, *68*, 052104.

[S17] Solozhenko, V. L., Kurakevych, O. O., Andrault, D., Le Godec, Y. & Mexouar, M. Ultimate metastable solubility of boron in diamond: synthesis of superhard diamondlike BC_5. *Phys. Rev. Lett.*, **102**, 015506 (2009).

[S18] Zarechnaya, E. Yu. et al. Superhard semiconducting optically transparent high pressyure phase of boron. *Phys. Rev. Lett.*, **102**, 185501 (2009).

[S19] J. Qin, N. Nishiyama, H. Ohfuji, T. Shinmei, L. Lei, D. He, T. Irifune, *Scripta Mater.*, **2012**, *67*, 257.
